# Time-resolved decoding of metabolic signatures of *in vitro* growth of the hemibiotrophic pathogen *Colletotrichum sublineolum*

**DOI:** 10.1038/s41598-019-38692-7

**Published:** 2019-03-01

**Authors:** Fidele Tugizimana, Arnaud T. Djami-Tchatchou, Johannes F. Fahrmann, Paul A. Steenkamp, Lizelle A. Piater, Ian A. Dubery

**Affiliations:** 1 Centre for Plant Metabolomics, Department of Biochemistry, University of Johannesburg, Auckland Park, Johannesburg, South Africa; 2 Department of Clinical Cancer Prevention, University of Texas MD Anderson Cancer Centre, 1515 Holcombe Blvd., Houston, TX 77030 USA

## Abstract

Metabolomics has emerged as a powerful approach to comprehensively interrogate cellular biochemistry. As such, we applied an untargeted liquid chromatography-mass spectrometry metabolomic strategy to elucidate metabolome changes in the anthracnose-causing hemibiotrophic sorghum pathogen, *Colletotrichum sublineolum*. An *in vitro* batch culture study model with different carbon sources, glucose, arabinose and rhamnose, were used to support fungal growth over a period of twelve days. Metabolites representing the intracellular and extracellular (secreted) metabolomes were extracted with methanol and subjected to LC-MS analyses. Chemometric modelling revealed a metabolic variation trajectory, comprising three distinct stages that metabolically describe the adaptation of the fungus to diminishing nutrients. Selected marker gene expression indicated stage one (0–3 d.p.i) as corresponding to the early logarithmic phase. Stage two can be interpreted as an intermediate transitionary stage with stage three corresponding to the stationary phase (9–12 d.p.i). Stage one was characterised by up-regulation of endo-metabolites such as ferricrocin, fatty acids and flavone-conjugates, while stage three was characterised by the secretion of phytotoxins, including colletotrichin and colletotric acid. Ultimately, results from our *in vitro* model reveal previously unknown insights into the dynamic aspects of metabolome reprogramming in the growth phases of *Colletotrichum* spp as determined by nutrients obtainable from plant cell walls.

## Introduction

*Colletotrichum* is one of the most widespread and economically detrimental genera of plant pathogenic fungi, and represents a serious threat to global food security and ecosystem health^1,2^. Members of this genus are etiological agents of anthracnose leaf spot and other diseases including blights and post-harvest rots on a vast range of agronomic and horticultural crops^3,4^. The severe damage caused by *Colletotrichum* spp leads to major losses in economically important crops, extending particularly to vital staple foods in tropical and sub-tropical regions^4,5^.

*Colletotrichum* pathology characteristically follows a multistage hemibiotrophic infection strategy involving the formation of a series of specialised cell types^6^. Following adhesion of spores, melanin-pigmented appressoria are formed and mediate initial host penetration using mechanical force and enzymatic degradation. Furthermore, *Colletotrichum* fungi differentiate specialised infection vesicles, which then begin the growth of primary hyphae that develop inside living host cells surrounded by an intact host plasma membrane^6,7^. This symptomless stage of infection, *i.e*. biotrophy, is short lived and the fungus switches to a necrotrophic stage, forming differentiated and morphologically distinct secondary hyphae that kill and destroy host tissue for nutrient acquisition. The lifestyle transition involves dynamic multi-layered reprogramming, including the expression of a vast array of genes encoding lytic enzymes, transporters and secondary metabolism-related enzymes as well as toxin biosynthesis that accompanies rapid growth, systemic colonisation and breakdown of host cells^6–9^.

Extensive studies have been carried out using cytological, physiological, genomic and transcriptomic approaches to elucidate the molecular details that govern the lifestyles of hemibiotrophic *Colletotrichum* fungi, and have provided substantial knowledge of the underlying infection strategies^6–10^. Furthermore, considering high diversity and tractability to *in vitro* culture, *Colletotrichum* spp provide excellent models for studying the molecular mechanisms and biochemistry of infection structure differentiation and fungal-plant interactions^11,12^. However, there is still much to uncover about the chemical signalling and regulatory mechanisms underlying the transition from biotrophy to necrotrophy of *Colletotrichum* pathogens that may allow the development of novel approaches to control fungal diseases in crop plants.

Studies of fungal growth *in planta* are technically challenging and represents a specific challenge to metabolomic approaches due to the close association between plant and pathogen, coupled with a general commonality of metabolites^13^. Here, we opted for an *in vitro* culture system, coupled to a liquid chromatography-mass spectrometry (LC-MS)-based untargeted metabolomic approach to gain information about the underlying biochemistry associated with the different growth stages of *C. sublineolum*. This was achieved by profiling the methanol soluble secondary - and some primary metabolites present in the endo- and exometabolomes that characterise the different stages of growth in batch culture. The uncovered metabolic signatures, accompanied with expression levels of selected marker genes, identified specific biochemical processes involved in the growth stages of the fungus, generating a holistic molecular description of the trophic transition phenomenology.

## Results and Discussion

### Growth of *C. sublineolum* on different carbon sources

Understanding nutrient acquisition modes employed by hemibiotrophic phytopathogenic fungi, with carbon metabolism as an essential feature^14^, can provide informative insights about the biochemical underpinnings governing fungal adaptation to the host environment^15^. Glucose, polymers of glucose and related derivatives are main constituents of plant cell walls and are also present in the apoplast and intracellular compartments^16,17^, while arabinose and rhamnose are neutral sugars in the pectic polysaccharide fraction^15,18,19^. Previous studies have indicated that arabinose and rhamnose stimulate endopolygalacturonase gene expression in *C. lindemuthianum*, important for establishing the fungal infection by targeting the pectin polymers in the cell wall and middle lamella^15^. Here, following a reductionist approach, different carbon sources namely glucose, arabinose and rhamnose^15^ were added to Murashige and Skoog (MS) culture medium to provide a controlled *in vitro* nutrient environment for *C. sublineolum* growth. This basal salt medium was selected as it does not contain any organic compounds as nutrients that could interfere with subsequent downstream metabolomics analyses. *C. sublineolum* could grow in the culture media containing these different carbon sources (with a slow rate in rhamnose-containing media)^15^ with a noticeable carbon assimilation (Supplementary Fig. 1A–D) which reflects the nutrition-suitability thereof.

### Metabolomic profiling of *C. sublineolum* growth stages

**Figure 1:**
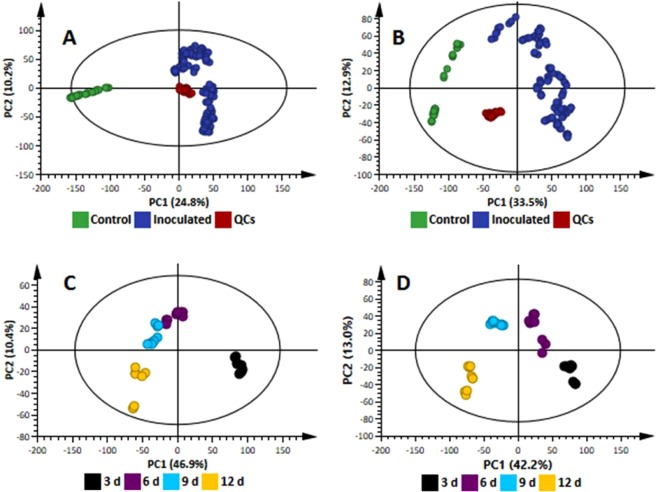
Principal component analysis (PCA) scores plots of the UHPLC-MS data. (**A,B**) Infographically display the assessment of the quality of the acquired data. (**A**) A 12-component model (R^2^ = 0.721 and Q^2^ = 0.624) of ESI positive data (Pareto-scaled) from extracellular samples (all three carbon sources). The control samples refer to the non-inoculated media. The quality control (QC) samples cluster together, which is an indication of good quality of the acquired data. (**B**) A 14-component model (R^2^ = 0.787 and Q^2^ = 0.708) of ESI negative, Pareto-scaled data from extracellular samples (all three carbon sources). QC samples cluster together with almost no within-group variation. (**C**) A PCA scores scatter plots of the PCA model of X-data (ESI negative) from extracellular extracts of *C. sublineolum* grown on arabinose: a 2-component model, explaining 59.5% of the total variation in the Pareto-scaled data, with the amount of predicted variation by the model, according to cross-validation, as 53.9%. (**D**) A PCA scores scatter plot (first two components) of X-data (ESI negative) from intracellular extracts of *C. sublineolum* grown on arabinose: a 3-component model, with R^2^ = 0.603 and Q^2^ = 0.51, of Pareto-scaled data X. In both (**C**,**D**), data from each time point form differential groups (no within-group variation) and a clear time-related trend is observed. These clear time-related differences and time-course trends were also observed in data sets from glucose and rhamnose carbon sources and different ESI modes, as indicated in Supplementary Figs 8 and 9.

Both intra- and extracellular (secreted into the medium) methanol-soluble metabolite extracts were analysed on an LC-MS analytical platform, allowing the simultaneous detection of multiple analytes (primarily mid-polar metabolites that, in addition to secondary metabolites, included some primary metabolites) with high sensitivity. Chromatographically, the resultant differential profiles (Supplementary Figs 2–7) provided a visual indication of changes in extracted metabolites occurring over time, and point to alterations in the metabolome during growth and development. To further elucidate the functional readouts of cellular physiological states related to the fungal growth, chemometric analyses were applied to the collected LC-MS data. Following data pre-processing, principal component analysis (PCA) was firstly applied to summarise the multidimensional data in an intelligible way that grasps the silent characteristics of the data. Thus, PC analyses allowed descriptive assessment of the distribution of samples so as to evaluate the quality of the data and detect natural groupings, trends and outliers. The infographic output of the PCA modelling showed, in the scores space, no strong outliers (observations that do not fit the model, *i.e*. outside the 95% confidence interval, ellipse). The PCA modelling indicated stability, reliability and reproducibility of the analyses – as confirmed by the quality control (QC) samples clustering closely in the center of the plot –, and natural separation between control and inoculated samples (Fig. 1A,B). Furthermore, PCA models revealed distinct time-related groupings in both the endo- and exo-‘metabolite space’ in all carbon source conditions. Here, clear sample groupings indicate dynamic changes occurring in *C. sublineolum* metabolism during growth in the culture media, and an underlying time-trend that characterises these metabolic changes (Fig. 1C,D and Supplementary Figs 8 and 9).

As mentioned, once host cells have been penetrated, *Colletotrichum* spp deploy multistage mechanisms that are crucial for fungal establishment and successful infection. Although there is still considerable uncertainty with regard to differentiation between nutrients obtained directly from the host and that which is *de novo* synthesised by the invading pathogen, it is known that *Colletotrichum* fungi employ different nutrient acquisition mechanisms^11,18,20^. These strategies are associated with dynamic developmental processes and may be viewed as time-dependent stages, related by the time trend(s) revealed by PC analyses (*i.e* time-related sample groupings in scores space) (Fig. 1 and Supplementary Figs 8 and 9). However, PCA, as the standard model to deal with high-dimensional and complex metabolomic data sets, is mathematically a non-dynamic method, due to its insensitivity to the evolutionary nature of the time axis. Standard PC-based analyses can only uncover time-trends in a data set, without necessarily efficiently defining these time-related trajectories^21,22^. Hence, in this study, batch statistical processing or modelling (BP/M)^22,23^ was utilized to descriptively explain these time-related metabolic changes in *C. sublineolum* growth revealed by PCA, treating each carbon source (glucose, arabinose and rhamnose) as an individual batch, comprising a series of times (3, 6, 9, 12 d.p.i. samples). BP/M provided an efficient means of infographically visualising the biochemical response to the type of carbon source in terms of both inter-group variation and net variation in intra/extracellular metabolite profiles. BP/M allowed for the evolution of the metabolic changes to be statistically characterised and described in time trajectories and provided thus a template for defining the sequence of time-dependent metabolic profiles. Significant time-related changes could subsequently be identified, as well as metabolic features (metabolite signals) related to these changes.

The three-way data matrix built up by the batches (carbon source conditions), the pre-processed LC-MS variables (*m/z*, Rt variables: spectral descriptors) and the metabolite collection time (the maturity variable) was decomposed by subjecting it to two subsequent levels of multivariate analysis: batch evolution (or lower level) and batch level (or upper level) modelling^22–24^. The former is based on PLS regression against metabolite collection time, thus providing a descriptive characterisation of the metabolic variation trajectory in terms of PLS components. PLS takes advantage of the correlation that already exists between the LC-MS variables (spectral descriptors) and time; generating scores plots that explain the greatest variance in the data with respect to time.

**Figure 2:**
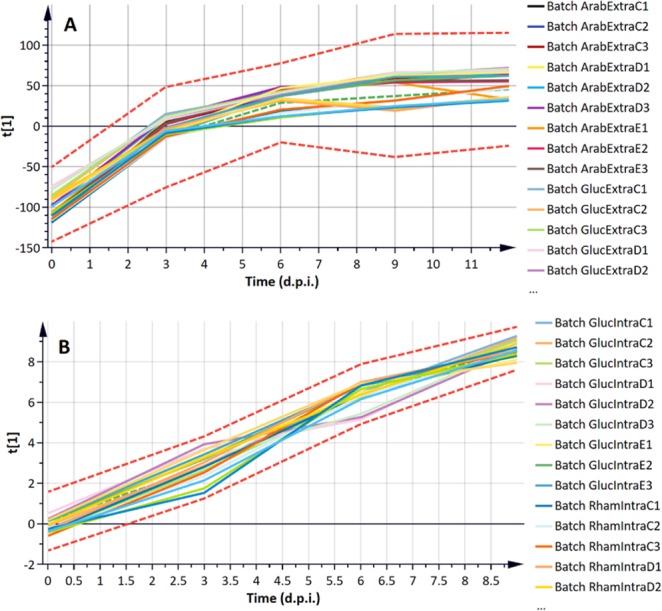
Partial least squares (PLS) trajectory plots from batch evolution modelling of ESI negative data: samples from *C. sublineolum* grown on different carbon sources (arabinose, glucose and rhamnose) for 3, 6, 9 and 12 d.p.i. (**A**) Extracts from extracellular samples – the computed lower-level PLS model of three significant components explaining 63.7% of the variation in the Pareto-scaled data X and 99.1% of the variation in the response Y (time). The predicted variation by the model, according to cross-validation, is 98.6%, indicating a significantly substantial relationship with time (CV-ANOVA *p*-value = 3.8 × 10^−30^). (**B**) Extracts from intracellular samples – a 4-component PLS model explains 56.9% of the variation in the Pareto-scaled data X (from intracellular samples), and 91.9% of the variation in the response Y (time). The predicted variation by the model = 90.1%, and CV-ANOVA *p*-value = 0. In both (**A**,**B**) the green dotted line indicates the average and the red dotted lines the ±3 SD. Each line represents a sample from a batch; and a batch refers to a carbon source. Data from all carbon sources (glucose, arabinose and rhamnose) are computed together. Due to figure size not all names/labels are detailed. The computed time-related evolution thus provides a descriptive characterisation of time-dependent metabolic changes.

The computed batch evolution (lower level PLS) models were statistically significant and reliable, with a good predictability in **Y** (time), according to cross-validation, and indicated a significantly substantial relationship of **X**-variables with time (Fig. 2 and Supplementary Fig. 10A). Since the first vector ***t***[1] is the vector direction that normally explains the maximum covariance between **X** and **Y**, plots of the first scores vector (***t***[1]) were mapped to generate a time-course trajectory plot describing the different phases of the dominant metabolic perturbations with time (Fig. 2). Lower scores vectors (***t***[2]-***t***[n]), which progressively explained less of the covariance in the data and orthogonal to each other, were also plotted over time to establish more subtle, time-related metabolic variations (Supplementary Fig. 10B). The batch time-evolution for the fungal growth (data from extracted endo- and exo-metabolite samples) together with the average trajectory (dotted green line) and the ±3 SD control limits (dotted red lines) indicated a significant increase in scores 3 d.p.i., as described by the first PLS scores vector ***t***[1] (Fig. 2). Thereafter, a relatively smoother increase in ***t***[1] followed up to 6 d.p.i. The evolution kept increasing (with slightly changing slope) until the last time point, and no deviating behaviour (outside the ±3 SD control limits) was observed.

**Figure 3:**
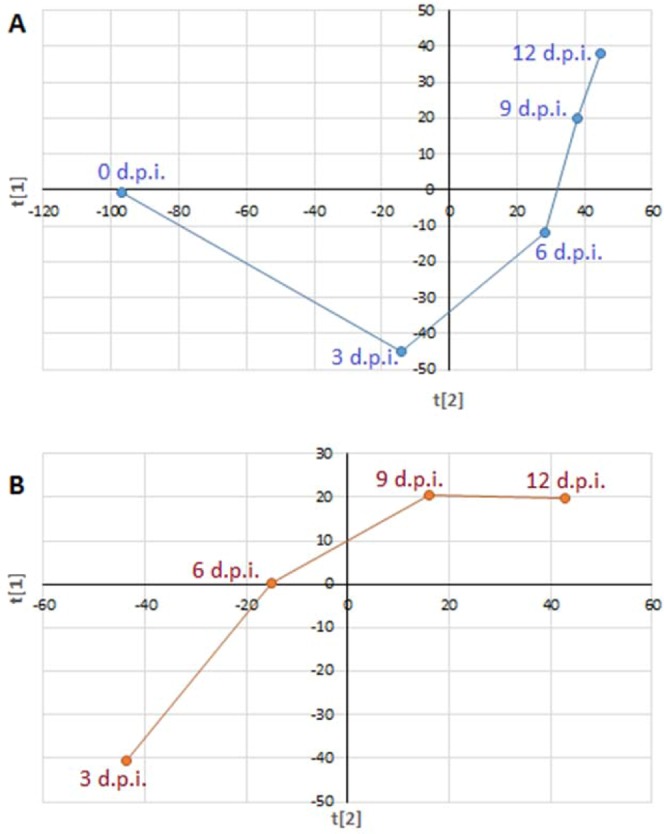
Aligned average partial least squares (PLS) trajectory plots *t*[1]/*t*[2]. (**A**) Represents extracellular samples and (**B**) intracellular samples, (data from all carbon sources and ESI negative mode MS). Since on 0 d.p.i. there was no sufficient fungal material yet for intracellular metabolite extraction, the intracellular data (**B**) start on 3 d.p.i. The plots show three distinct phases corresponding to the time periods: 0–3 (stage 1), 3–6 (stage 2), and 9–12 d.p.i. (stage 3).

Furthermore, the (aligned) average PLS trajectory plotted in two dimensions (*t*[2]/*t*[1] vectors) (Fig. 3) clearly indicates that the modelled metabolic evolution comprised three distinct and significant phases corresponding to the time periods of 0–3, 3–6 and 6–12 d.p.i. These uncovered intervals of metabolic reprogramming point to distinct stages of *C. sublineolum* growth, which can be postulated to be early adaptation (0–3 d.p.i.), transition (3–6 d.p.i.) and stationary (6–12 d.p.i.). These mathematically elucidated developmental time-periods of fungal growth correlate to previous studies, which reported that the biotrophic phase of *Colletotrichum* pathogens is short-lived, lasting about 72 h post-infection/inoculation, followed by the switch to necrotrophic lifestyle^6,10–12^. However, very little substantial knowledge exists with regards to the underlying biochemical and molecular mechanisms and differences in growth stages do not necessarily correspond to biotrophy or necrotrophy. Hence, it could be possible that there is actually a transition period (as this *in vitro* study suggests: 3–6 d.p.i.) that can inform on the *in planta* situation where the fungus gradually responds to environmental cues and adapts to a complete necrotrophic phase.

### Differential gene expression profiling indicative of metabolic reprogramming in *C. sublineolum*

**Figure 4:**
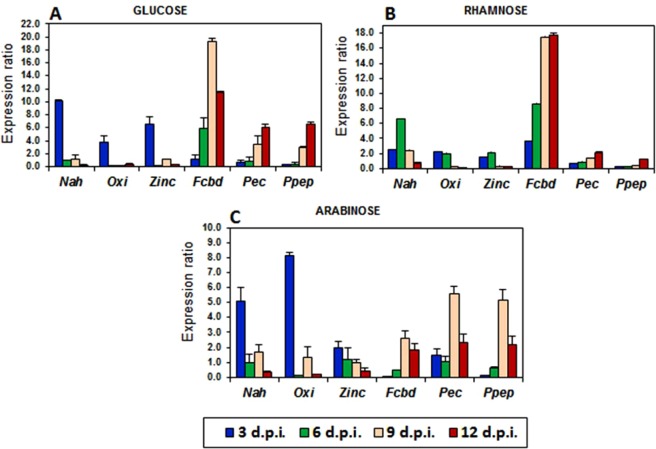
Gene expression analysis of group 1 and −2 gene markers in *C. sublineolum* grown in MS media containing either glucose (**A**), rhamnose (**B**) or arabinose (**C**). The data was normalised using the *actin* gene to give the relative gene expression wherein error bars represent the standard error of mean. The marker genes for stage 1 were: *Zinc carboxypeptidase (Zinc), putative sodium/hydrogen exchanger family protein (Nah), GMC Oxidoreductase (Oxi)*; and for stage 3: *fungal cellulose binding domain-containing protein (Fcbd), pectate lyase (Pec) and putative peptidase family M28 (Ppep)*.

To confirm these chemometrically characterised phases as corresponding to distinct metabolomes, quantitative expression analyses of selected marker genes were carried out, with *actin* as a reference gene. Quantitative measurements were normalised to give the relative gene expression wherein error bars represent the standard error of mean (SEM) (Fig. 4).

The group 1 marker genes, reported to be associated with a biotrophic phase in related *Colletotrichum graminicola*^6^, namely *sodium/hydrogen exchanger family protein* (*NaH*), *GMC oxidoreductase* (*Oxi*) and *zinc carboxypeptidase* (*Zinc*), showed a significant up-regulation at 3 d.p.i., independently of carbon source, where after the expression levels decreased from 6 d.p.i., with a significant decrease in expression levels at 12 d.p.i. (Fig. 4). However, some carbon source-related nuances could be observed as in rhamnose-containing culture media, where the expression levels of these genes were high even at 6 d.p.i. and significantly decreased at 9 d.p.i. (Fig. 4B). In contrast, the group 2 marker genes, expressed during a necrotrophic phase^6^, namely *fungal cellulose binding domain-containing protein* (*Fcbd*), *pectate lyase* (*Pec*) and *putative peptidase family M28* (*Ppep*) were significantly expressed at 9–12 d.p.i. (Fig. 4), independently of the culture milieu. Nonetheless, the expression levels of *Pec* and *Ppep* genes were higher than that of the *Fcbd* gene in *C. sublineolum* cultured in arabinose-containing media (Fig. 4C). In both glucose- and rhamnose-containing media, on the other hand, the *Fcbd* gene was the highest expressed group 2 gene (Fig. 4A,B).

Despite some carbon source-related differential gene expression levels observed, the results indicate that the 0–3 d.p.i. time interval is associated with group 1 gene expression, whereas group 2 gene expression is linked to the time period of 9–12 d.p.i. (Fig. 4). Although the expression levels of selected group 2 marker genes were observed to be significant at 9 d.p.i. (e.g. *Fcbd* gene), the expression of these genes could be seen also at 6 d.p.i. (Fig. 4). Except for rhamnose-containing media (in which the group 1 marker genes were expressed at 6 d.p.i. – Fig. 4B), the time interval between 3 and 6 d.p.i. could be related to the transition phase. These observations confirm the chemometrically identified metabolic phases (Figs 2 and 3) as corresponding to distinct metabolomes (0–3 d.p.i.) *vs*. (9–12 d.p.i.) during the *in vitro* growth, development and differentiation of *C. sublineolum* in batch culture.

### Metabolic signatures associated with early – *vs*. late trophic stages of *C. sublineolum*

The putatively identified metabolites, from both endo- and exo-metabolomes, statistically extracted to be discriminant for the two dominant trophic stages (0–3 d.p.i. *vs*. – 9–12 d.p.i.), were of diverse metabolic origins and biochemical functions during fungal growth (Table 1). The metabolite profile of stage 1 (chemometrically described as the 0–3 d.p.i. time interval, Fig. 3) was characterised by up-regulation of intracellular metabolites (3 d.p.i./6 d.p.i.-fold change ratios >1) with a range of biochemical functions. Similarly, fold change ratio values > 1 of 12 d.p.i. *vs*. 3 d.p.i. were used to obtain information on metabolites associated with stage 3 (Table 1). Both primary as well as secondary metabolites were associated with stage 1as well as stage 3. Interestingly, with the exception of arachidonoyl amine and indole-3-acetyl-glutamic acid, the fold change ratios of the rhamnose *vs*. glucose comparison, fell within a ± 2 range.

**Table 1:** Summary of annotated metabolites that contributed to the discriminating variability in the altered metabolomes of *Colletotrichum sublineolum* grown on glucose, arabinose and rhamnose, as described by chemometric models.

#	Metabolite	*m/z*	Rt (min)	Ion mode	Ion	Molecular formula	Fold change			Milieu	Biochemical Class
							3 d/6 d^*^	12 d/3 d^#^	Rh/Glc^$^		
1	**Lumichrome (7,8-dimethylalloxazine)**	285.0371	13.69	neg	[M + Na_Na-H]^−^	C_12_H_10_N_4_O_2_	74.6	7.5	0.6	Endo-	Flavin
2	**Ferricrocin-iron siderophore**	771.2507	7.19	pos	[M + H]^+^	C_28_H_44_FeN_9_O_13_	12.4	4.5	0.6	Endo-	Siderophore
3	**2-Phenylbutyric acid**	187.0729	9.74	pos	[M + Na + H]^+^	C_10_H_12_O_2_	4.9	4.2	0.7	Endo-	Organic acid
4	**2,15,16-Trihydroxy palmitic acid**	325.1991	14.69	neg	[M + Na-H]^−^	C_16_H_32_O_5_	34.2	3.1	1.4	Endo-	Fatty acid
5	**Hydroxy-acetyl-cysteinyl-dihydronaphthalene**	376.0823	9.66	pos	[M + HCOONa]^+^	C_15_H_17_NO_4_S	4.4	2.2	1.1	Endo-	Naphthalene
6	**Trihydroxy-methoxyanthraquinone**	287.0545	9.63	pos	[M + H]^+^	C_15_H_10_O_6_	5.1	1.9	0.6	Endo-	Anthraquinone
7	**Malonamoyl-CoA**	870.1695	6.18	pos	[M + NH4]^+^	C_24_H_39_N_8_O_18_P_3_S	2.2	0.9	0.6	Endo-	Fatty acyl CoA
8	**Coprogen hydroxamate siderophore**	822.313	9.14	pos	[M + H]^+^	C_35_H_53_FeN_6_O_13_	8.6	0.8	1.1	Endo-	Siderophore
9	**Arachidonoyl amine**	346.2114	13.49	neg	[M + Na_Na-H]^−^	C_20_H_33_NO	13.2	0.8	14.6	Endo-	Fatty amide
10	**3-Hydroxy-2-oxindole-3-acetyl-asp**	343.0555	13.02	neg	[M + Na-H]^−^	C_14_H_14_N_2_O_7_	5.2	0.8	0.1	Endo-	Indole
11	**Phosphatidylserine**	386.1202	5.18	pos	[M + H]^+^	C_13_H_24_NO_10_P	2.6	0.7	0.6	Endo-	Phospholipid
12	**Nonanoic acid (pelargonic acid)**	159.1373	9.97	pos	[M + H]^+^	C_9_H_18_O_2_	5.2	0.5	1.5	Endo-	Fatty acid
13	**9-Hydroxy-hexadecan-1,16-dioic acid**	301.2032	14.83	neg	[M − H]^−^	C_16_H_30_O_5_	13.8	0.5	0.2	Endo-	Dioic acid
14	**Fructoselysine 6-phosphate**	389.1326	8.97	pos	[M + H]^+^	C_12_H_25_N_2_O_10_P	9.1	0.5	0.7	Endo-	Intermediate
15	**Indole-3-acetyl-glutamic acid**	325.0796	12.60	neg	[M + Na-H]^−^	C_15_H_16_N_2_O_5_	12.2	0.2	6.8	Endo-	Indole
16	**Heptadecane**	239.2749	12.38	neg	[M − H]^−^	C_17_H_36_	30.5	0.1	1.6	Endo-	Alkane
17	**D-Glucose 6-phosphate**	259.0227	15.28	neg	[M − H]^−^	C_6_H_13_O_9_P	56.6	0.1	0.1	Endo-	Intermediate
18	**3′,5-Dihydroxy-3,4′,7-trimethoxyflavone**	343.0815	13.86	neg	[M − H]^−^	C_18_H_16_O_7_	11.3	0.0	0.7	Endo-	Flavanone
19	**5-Methoxysterigmatocystin**	377.0615	8.85	pos	[M + Na-H]^+^	C_19_H_14_O_7_	3.7	0.2	1.2	Endo-	Mycotoxin
20	*Colletotrichin*	491.327	11.78	pos	[M + H]^+^	C_28_H_42_O_7_	1.9	79.6	1.0	Exo-	Mycotoxin
21	*6-Demethylsterigmatocystin*	331.0236	15.19	neg	[M + Na-H]^−^	C_17_H_10_O_6_	0.2	55.5	0.7	Endo-	Mycotoxin
22	*Colletotric acid*	523.284	16.89	neg	[M − H]^−^	C_28_H_28_O_17_	2.0	49.9	0.1	Exo-	Mycotoxin
23	*Unidentified (sterigmatocystin related)*	373.0104	15.79	neg	[M − FA]^−^	C_17_H_12_O_7_	0.1	39.0	0.8	Exo-	Mycotoxin
24	*Pyridoxyl-glutamic acid-monophosphate*	377.0747	15.45	neg	[M − H]^−^	C_13_H_19_N_2_O_9_P	0.2	18.1	1.6	Endo-	Co-enzyme
25	*Mycophenolic acid*	321.2602	19.03	pos	[M + H]^+^	C_17_H_20_O_6_	0.7	5.6	0.2	Exo-	Antibiotic
26	N-Benzyl-4-sulfamoyl-benzamide	311.0474	15.80	neg	[M + Na-H]^−^	C_14_H_14_N_2_O_3_S	0.7	4.1	0.8	Endo-	Benzenoid
27	6-Hydroxy-5-methoxyindole glucuronide	338.0869	15.04	neg	[M − H]^+^	C_15_H_17_NO_8_	0.3	1.6	0.8	Endo-	Indole derv
28	5-Nitrofurfural	142.013	8.02	pos	[M + H]^+^	C_5_H_3_NO_4_	0.6	1.5	1.6	Endo-	Heterocyclic
29	N-acetyl-D-glucosaminitol	224.1139	8.74	pos	[M + H]^+^	C_8_H_17_NO_6_	1.4	1.1	1.9	Endo-	Fungal cell wall
30	2-Octenedioic acid	173.0813	9.06	pos	[M + H]^+^	C_8_H_12_O_4_	0.8	1.0	1.5	Endo-	Fatty acids

These discriminating metabolites were identified based on partial least squares (PLS) loadings plots (Supplementary Fig. 11) and reported to the Metabolomics Standards Initiative, level 2.

^*^Fold change computed by 3 d *vs*. 6 d for a quantitative assessment of stage 1.

^#^Fold change computed by 12 d *vs*. 3 d for a quantitative description of stage 3.

^$^Fold change computed by rhamnose *vs*. glucose for a quantitative assessment of the differential metabolic profiles in the two different carbon source media.

Bold: metabolic markers for stage 1; *Italic* = metabolic markers for stage 3; Black = metabolite markers that were less conclusive.

MSI-2 = Metabolomics Standards Initiative, level 2.

**Figure 5:**
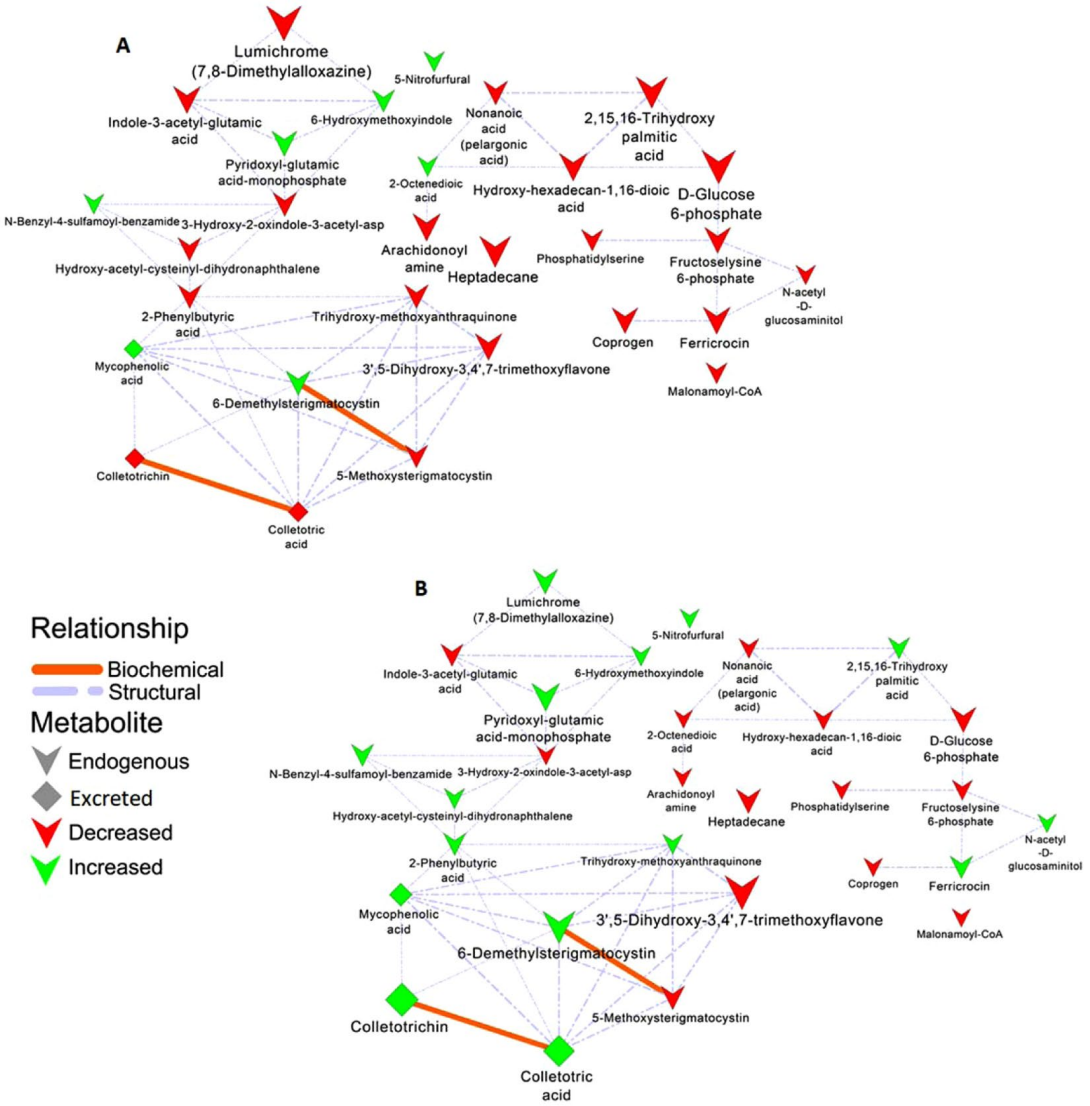
Metabolic network analysis: A biochemical and empirical network displaying metabolic relationship patterns between chemometrically selected metabolites – signatory biomarkers and metabolic phenomenologies for stage 1 (**A**) and stage 3 phases (**B**). Edge type and thickness depict the relationship (biochemical, structural) and Tanimoto correlation coefficient (structural only, with a cut-off of 0.5) between respective nodes. Node shape illustrates whether the metabolite is endogenous or secreted in the medium. Node colour and size reflect the direction (red – decreased; green – increased) and magnitude of change (fold-change of 3/6 d.p.i **(A)** and 12/3 d.p.i. (**B**)). The graph was visualized using Cytoscape version 3.5.1.

To gain more insights into possible biochemical and molecular frameworks that choreograph the transition of *C. sublineolum* growth from stage 1 to stage 3, a network analysis approach was carried out, using a high degree of correlation (biochemical and empirical) between the measured significant metabolites. The constructed correlation-based networks (Fig. 5) depict relational patterns in the experimental data (based on the fold changes listed in Table 1) and identify altered graph neighbourhoods which do not depend on any predefined biochemical pathways, thus allowing the characterisation of the molecular and cellular states induced by pathway interconnections under the stated experimental conditions.

Lumichrome (7,8-dimethylalloxazine), a breakdown product of riboflavin^25^, was found to be prominent marker during the active growth of stage 1. The levels subsequently decreased before increasing again during the nutrient-limiting stage stage 3 (Table 1, Fig. 5). The metabolite was reported to participate in aspects of symbiosis (reconfiguration of the host primary carbon and phytohormone metabolism) and enhancement of hyphae initiation^25^. The significant and dynamic changes in the level of this metabolite suggests the flavin-related metabolic pathways are activated during the early growth phase of *C. sublineolum*, with metabolites involved in establishment of a parasitic relationship between fungus and host. To orchestrate such a complex manipulation of the cellular trafficking and organisation in the host cell for successful biotrophy^8,12,20^, the fungus needs to reprogram its metabolism, deploying a coordinated and efficient transfer system for nutrient acquisition (*e.g*. involving flavin-related metabolism as this study suggests).

Furthermore, the stage 1 metabolite profile was characterised by hydroxylated free fatty acids, - amide and - dioic acid conjugates and derivatives (Table 1, Fig. 5). Scientific attention into the dynamics of the ‘lipidome’ during plant-fungal interactions has revealed that fungal lipids are involved in organism development as well as regulatory machinery for secondary metabolism. In this regard, linolenic -, oleic -, nonanoic - and decanoic acids have been shown to participate in the regulation of spore development and mycotoxin production in *Aspergillus* spp, *Fusarium* spp, and *Laetisaria arvalis*^26–28^. Moreover, nonanoic acid (pelargonic acid) is reported to weaken the waxy cuticle of plants, causing cell disruption and cell leakage (PubChem database, https://pubchem.ncbi.nlm.nih.gov). These properties might contribute to the establishment of a *C. sublineolum* infection and penetration associated with its biotrophic phase.

Important signatory biomarkers related to the early growth phase were the siderophores, namely ferricrocin and coprogen (decreasing in relative content from 3 d.p.i. to 6 d.p.i., Table 1, Fig. 5), which are essential molecules for iron uptake and storage^29–32^. The transition element, iron, is essential in key metabolic processes^30,31^ and plays a vital role in plant-microbe interactions^29,33,34^. A study on the related hemibiotrophic maize pathogen *C. graminicola*, revealed that siderophores are required for the establishment of vegetative growth of the fungus, and are also involved in modulating the plant immune system^35^.

Notably, the polyketide, 5-methoxysterigmatocystin, (Table 1, Fig. 5) was found as a marker for stage 1 growth (3.7-fold decrease from 3 d.p.i. to 6 d.p.i.). Sterigmatocystin is a highly toxic mycotoxin and initially reported from *Aspergillus* spp^26,36–39^. The intracellular occurrence of this methoxylated form of sterigmatocystin in *C. sublineolum* points to the initiation of reprogramming of secondary metabolism during stage 1, characterised by the biosynthesis of toxins to be deployed during nutrient-limiting conditions as during stage 3 (6-demethylsterigmatocystin). This finding is consistent with previous studies that indicated that the biotrophic phase of *Colletotrichum* spp is marked by activation of a genomic program involved in secondary metabolism, directed also to toxin production^6,8,10,20^.

The toxins that were found in this study to be secreted by *C. sublineolum* (during stage 3, chemometrically elucidated as 9–12 d.p.i. time period – Fig. 3) include colletotric acid and colletotrichin, among others (Table 1, Fig. 5). The production and secretion of these phytotoxins (mostly non-host-specific) by *Colletotrichum* spp have been reported to be a marker for necrotrophic development. During this stage, *Colletotrichum* spp satisfy nutritional needs by actively killing the host tissue *via* phytotoxin production or generation of reactive oxygen species mechanisms^6,10,20,40,41^.

It can be postulated that with the significant depletion of carbon sources (Supplementary Fig. 1), the fungus resorts to destructive mechanisms, characterised by the production of phytotoxins, for survival and proliferation. Such conjecture can be extrapolated to the *in planta* scenario. Furthermore, the generated networks topologically revealed clear modules: such as clusters/modules related to lumichrome, fatty acids, ferricrocin and colletotrichin (Fig. 5). These clusters point to underlying metabolic processes that define a transition from stage 1 to stage 3, suggesting highly complex and tightly regulated dynamic metabolism as the fungus transits from nutrient abundance to scarcity. The interweavement of different classes of significant metabolites within and between these different clusters thus describes silent features in this dynamic metabolism: coordinated regulations in both early - (*e.g*. changes in primary metabolism, fatty acid derivatives and siderophores) and late growth stages (exemplified by up-regulation and secretion of toxins). This observation correlates to a highly complex and coordinated genetic and transcriptional program, as well as capacity deployed by *Colletotrichum* in its infection strategy as previously reported; involving activation of genes related to degradative enzymes, expression of secondary metabolism gene clusters, metabolite diversity *via* transcriptional regulation^6,8^. Thus, this predictive description proposes that the *Colletotrichum* lifestyle is metabolically more complex (temporally and spatially) than currently understood, spanning various metabolic pathways.

## Conclusion

For successful infection of host plants, hemi-biotrophic fungal pathogens like the *Colletotrichum* spp depend heavily on the establishment of effective nutrient acquisition strategies, *i.e*. a biotrophic phase followed by a necrotrophic phase. This *in vitro* study model, using different carbon sources (glucose, arabinose or rhamnose), obtainable *in planta* as cell wall- and pectin-derived nutrients, provided insights into the metabolic changes in *C. sublineolum*, suggesting specific metabolic features during the growth course of the fungus.

Our results chemometrically describe that metabolic reprogramming occurs in *C. sublineolum* during the transition from stage 1 to stage 3 growth. By applying a dynamic multivariate method, the time-course phases were elucidated over a period of 12 days. Stage 1 was seen to be short lived (0–3 d.p.i.), followed by a transition interval, stage 2 (3–6 d.p.i.), leading to stage 3 (9–12 d.p.i.). Stage 1 was accompanied with the expression of genes reported to be associated with biotrophy while stage 3 was associated with expression of genes associated with necrotrophy in *C. graminicola*^6^.

The metabolic profiles of these growth stages of the fungus were characterised by reprogramming of primary and secondary metabolism, with production of phytotoxins in stage 3. The metabolic states, as uncovered by both the endo- and exo-metabolome profiles, point to complex metabolomic changes that might be of functional significance to the interaction between the pathogen and host in an *in planta* situation.

The marker gene expression during stage 1 *vs*. stage 3 would imply that endogenous signals related to nutrient depletion are necessary and sufficient to trigger the changes in gene expression associated with necrotrophy. However, care should be taken in extrapolating the *in vitro* growth results to biotrophic – and necrotrophic stages in infected plant tissues. Also, this model, does not address the possibility that other signals, originating from fungal structures in the infected plant or from the plant itself, might play contributing roles in such a lifestyle switch. However, it does lay a solid foundation for further targeted metabolomic studies related to specific stages of fungal growth and development, *in vitro* or *in planta*.

Unravelling metabolite profile patterns underlying the *C. sublineolum in vitro* growth stages thus pointed to specific biochemical processes and deciphered regulatory mechanisms, providing a holistic description of growth and adaptation to diminishing nutrient conditions. Such knowledge contributes to enhance insight into the infection strategies of the fungal pathogen and the dynamic intricacies of the molecular interactions between *Colletotrichum* spp and plant targets. As such, the present work provides a necessary step for *in planta* studies, uncovering metabolic central hubs and key drivers in molecular events governing the *C. sublineolum* biology. Finally, the metabolomic information presented in this work will be a valuable resource for relating data from other omics layers towards systems biology description of the phenomenology of plant-fungal pathogen interactions. *Colletotrichum* spp represent economically significant pathogens where a holistic and detailed understanding of pathogen biology could substantially inform strategies for targeted disease control and management.

## Methods

### Fungal cultures, media and inoculation

A pathogenic isolate of *C. sublineolum* (PPRI 7183) was grown and maintained on potato dextrose agar (PDA). The working sub-cultures were maintained on half-strength PDA solid media in Petri dishes and incubated under a 12 h–fluorescent light cycle at 25 °C. To simulate *in planta* nutrient sources, three types of Murashige and Skoog (MS; without vitamins)-based liquid media (Duchefa, Haarlem, Netherlands, product number M0221) containing 10 g L^−1^ of different carbon sources, namely glucose, arabinose and rhamnose, were prepared under sterile conditions^42^.

One hundred mL Erlenmeyer flasks containing 50 mL of the MS liquid media (containing the various carbon sources) were each inoculated with three mycelial plugs (2 mm diameter) from *C. sublineolum* grown on solid PDA media (14 day-old cultures). The flasks were incubated at 25 °C on a rotary shaker at 130 rpm under a 12 h–fluorescent light cycle. Consumption of glucose during the growth stages of the fungus was quantitatively assessed using a glucose measuring device (Accu-Check^®^, Roche Diagnostics) on supernatants separated from the mycelial bio-mass harvested at 3, 6, 9 and 12 days post-inoculation (d.p.i.) (Supplementary Fig. 1A–D).

The experiment was conducted in three biological replicates. For extracellular samples, non-inoculated liquid media was also incubated and harvested under the same conditions, to account for any change that might have occurred in the media over time and served as negative controls.

### Extraction of intracellular and extracellular metabolites, and sample preparation

The organic solvents used, methanol and acetonitrile, were UHPLC-MS grade quality (Romil, Cambridge, UK), and water was purified by a Milli-Q Gradient A10 system (Millipore, Billerica, MA, USA). Leucine enkephalin and formic acid were purchased from Sigma Aldrich/Merck (Munich, Germany).

Each flask was harvested by filtering the contents through a double layer of Miracloth under vacuum to separate the fungal mycelia and the media. The mycelia samples were weighed and the wet-weight recorded. The extracellular metabolites (the exo-metabolome: metabolites secreted into the media) were extracted from 25 mL freeze-dried media filtrate with 50% methanol and filtered through a 0.22 µm nylon filter into glass chromatography vials fitted with 500 µL inserts. The intracellular metabolites (endo-metabolome) were extracted using 100% methanol added directly to the weighed-out fungal material in a ratio of 1:10 (w/v), and immediately homogenised using an Ultra Turrax homogeniser followed by sonication using a probe sonicator (Bandelin Sonopuls, Berlin, Germany), set at 55% power for 20 sec and repeated twice, which served to rapidly quench enzymatic activities^43,44^.

The homogenates were then centrifuged at 5000 × *g* for 15 min at 4 °C. The supernatants were placed in 50 mL round-bottom flasks and evaporated to 1 mL at 50 °C using rotary evaporation under vacuum, and dried to completeness with a speed vacuum centrifuge at 45 °C. The dried residues were re-constituted in 300 µL 50% methanol and filtered through a 0.22 µm nylon filter as described. The filtered aqueous-methanol extracts prepared from the endo- and exo-metabolomes were stored at −20 °C until analysed.

### Ultra-high performance liquid chromatography – mass spectrometry (UHPLC-MS) analyses

Chromatographic separation of metabolites was performed on an Acquity UHPLC system (Waters Corporations, Milford, MA, USA) using a conditioned autosampler at 4 °C. Two µL of methanol-extracted samples were separated on an analytical C18 column HSS T3 (150 mm × 2.1 mm, 1.7 µm – Acquity, Waters, Milford, MA, USA), thermostatted at 60 °C. Degassed solutions of formic acid: ultra-pure water (1:10^3^, v/v) (eluent A) and formic acid: acetonitrile (1:10^3^, v/v) (eluent B) were pumped at 0.4 mL min^−1^ into the UHPLC system. The applied gradient started at 5% B and increased linearly to 95% B over 22 min. The conditions were kept constant for 3 min to wash the column and returned to initial conditions at 27 min. The analytical column was allowed to equilibrate for 3 min. Each sample was analysed in triplicate to account for any analytical variability and the run time for each analysis was 30 min. The LC system was coupled to a SYNAPT G1 high definition (HD) quadrupole time-of-flight (Q-TOF) mass spectrometer (Waters, Milford, MA, USA), with an electrospray ionisation (ESI) source. The TOF analyser was used in V-optics, and the data were acquired in centroid mode.

Both positive and negative ionisation modes were employed with the scan range of 100–1000 Da, a scan time of 0.2 s and an inter-scan delay of 0.02 s. A lock spray source was used allowing online mass correction to obtain high mass accuracy of analytes (typically between 1 and 5 mDa mass accuracy). Leucine enkephalin, [M + H]^+^  = 556.2766 and [M − H]^−^ = 554.2615, was used as a lock mass reference, being continuously sampled every 15 s at a flow rate of 0.4 mL min^−1^, producing an average intensity of 350 counts scan^−1^ in centroid mode. The optimal conditions for MS analysis were as follows: source temperature: 120 °C; desolvation temperature: 450 °C; cone gas flow 50 L h^−1^; capillary voltage: 2.5 kV; sampling cone voltage: 17 V; extraction cone voltage: 4 V; desolvation gas flow: 550 L h^−1^. To assist with downstream structure elucidation and compound identification, the MS analyses were set to perform unfragmented as well as four fragmenting experiments (MS^E^) simultaneously by collision energy ramping from 10 to 40 eV. The software used to control the hyphenated system and perform all data manipulation was MassLynx 4.1 (SCN 704, Waters Corporation, Milford, MA, USA).

Quality control (QC) pooled samples were used to condition the LC-MS analytical system so as to assess the reliability and reproducibility of the analysis, and for non-linear signal correction^45,46^. Sample acquisition was randomised and the QC sample (6 injections) analysed every 10 injections to monitor and correct changes in the instrument response. Furthermore, 6 QC runs were performed at the beginning and end of the batch to ensure system equilibration. Such sample randomisation provides stochastic stratification in sample acquisition so as to minimise measurement bias. In the principal components analysis (PCA) space, the QC samples were clustered closely to each other (Fig. 1A,B), thus confirming the stability of the LC-MS system, and the reliability and reproducibility of the analyses.

### Data analysis: data set matrix creation and chemometric analyses

Visualisation and data processing were performed using MassLynx XS^TM^ 4.1 software (Waters Corporation, Manchester, UK) for both the centroid ESI positive and negative raw data. The MarkerLynx^TM^ application manager of the MassLynx software was used for data pre-processing (matrix creation), producing a matrix of retention time (Rt)-*m/z* variable pairs, with *m/z* peak intensity for each sample. The parameters of the MarkerLynx application were set to analyse the 1–25 min Rt range of the mass chromatogram, mass range 100–1000 Da, and alignment of peaks across samples within the range of ±0.05 Da and ±0.20 min mass and Rt windows, respectively; and mass tolerance of 0.01 Da. The MarkerLynx application uses the patented *ApexPeakTrack* algorithm to perform accurate peak detection and alignment. Following the peak detection, the associated ions are analysed (the maximum intensity, Rt and exact *m/z* mass) and captured for all samples. In this study, sample normalisation was done by using total ion intensities of each defined peak. Prior to calculating intensities, MarkerLynx performs a patented modified Savitzky-Golay smoothing and integration.

The data matrices thus generated were exported into SIMCA (soft independent modelling of class analogy) software, version 14 (Umetrics, Umeå, Sweden) for chemometric analyses, including multivariate and univariate statistical modelling, and an unsupervised method, namely PCA, was employed. The data pre-treatment methods used included Pareto-scaling, and as described in the results section, the computed models were rigorously validated.

A partial least squares (PLS)-based approach, namely batch processing/modelling (BP/M), was used to optimally assess and describe the time-related metabolic trajectories observed from PC-analyses^22,23^, allowing a multivariate statistical description of time-related events. In a BP/M approach, PLS regression analysis against time (*i.e*. the time period over which the changes are recorded) allows for the evolution of the metabolic changes to be statistically characterized and described in time trajectories. Thus, significant time-related changes can subsequently be identified, as well as metabolic features (metabolite signals) related to/that explain these changes^21,23,24^.

Biochemical networks were generated using Cytoscape^47,48^. Structural similarities were determined based on similarities between PubChem Substructure Fingerprints (ftp://ftp.ncbi.nlm.nih.gov/pubchem/specifications/pubchem_fingerprints.txt). The R package Chemminer generated molecular fingerprints using the PubChem Power User Gateway (PUG). Molecular fingerprints in the form of ordered lists of binary bits defining presence or absence of physical properties (*e.g*. element type, functional group, nearest neighbours) were used to calculate structural similarities; pairwise similarities were calculated based on Tanimoto similarity between two bit vectors resulting in scores between 0 and 1, where a score of 0 or 1 defines no or complete overlap in structural properties between the two molecules. A Tanimoto coefficient cut-off of 0.5 was used for network analyses.

### Metabolite identification

Data matrices from MarkerLynx^TM^-based data processing were exported to the Taverna workbench (www.taverna.org.uk) for PUTMEDID_LCMS Metabolite ID workflows^49^. The Taverna workflows consist of correlation analysis, metabolic feature - and metabolite annotation and allow for integrated, automated and high-throughput annotation and putative metabolite identification from LC-ESI-MS metabolomic data. The Taverna Metabolite ID procedure involves: (i) Pearson-based correlation analysis (*List_CorrData*), (ii) metabolic feature annotation (*annotate_Massmatch*) – allowing for grouping together ion peaks with similar features such as Rt, and annotating features with the type of *m/z* ion (molecular ion, isotope, adduct, others) believed to originate from the same compound. The elemental composition/molecular formula (MF) of each *m/z* ion was then automatically calculated; and (iii) metabolite annotation (*matchMF-MF*) of the calculated MF (from the output file from workflow 2) was automatically compared and matched to the MF from a pre-defined reference file of metabolites.

For confidence in metabolite annotation, the following steps were performed: (i) the calculated MF of a selected metabolite candidate was manually searched against databases and bioinformatics tools, such as the Dictionary of Natural Products (DNP) (www.dnp.chemnetbase.com), Chemspider (www.chemspider.com), Knapsack database (http://kanaya.naist.jp/KNApSAcK/) and KEGG (www.genome.jp/kegg/); (ii) structural confirmation through careful inspection of fragmentation patterns by examining the MS^1^ and MS^E^ spectra of the selected metabolite candidate. Metabolites were annotated to level 2 as classified by the Metabolomics Standard Initiative (MSI)^50^.

### Gene expression analyses - qPCR and data analysis

Total RNA was extracted from 100 mg mycelia of all time point samples using the Trizol-reagent (Invitrogen, Carlsbad, CA, USA) and then subjected to DNase treatment using DNase I (Thermo Scientific, Waltham, MA, USA), per the manufacturers’ instructions. Concentrations were determined using a NanoDrop® ND-1000^TM^ Spectrophotometer (NanoDrop Inc., Wilmington, DE, USA) and the integrity verified by denaturing agarose gel electrophoresis.

Based on the putative function during biotrophic and necrotrophic development of *C. graminicola*^6^, six *C. sublineolum* genes were selected for gene expression analysis in *C. sublineolum* grown in a culture containing either arabinose, glucose or rhamnose as carbon source. The selected marker-genes for the biotrophic phase were *zinc carboxypeptidase* (JMSE01000325.1), *putative sodium/hydrogen exchanger family protein* (JMSE01001189.1) and *glucose-methanol-choline (GMC) oxidoreductase* (JMSE01001299.1); while the genes for the necrotrophic phase included *fungal cellulose binding domain-containing protein* (JMSE01001209.1), *pectate lyase* (JMSE01001554.1) and *putative peptidase family M28* (JMSE01001300.1). The primer pairs (Supplementary Table 1) were designed using the ‘Primer Quest’ tool (Integrated DNA Technologies, Coralville, IA, USA) from the sequences obtained in Genbank (www.ncbi.nlm.nih.gov/genbank). Prior to quantification of the expression levels, the DNase-treated RNA was reverse-transcribed to cDNA using a RevertAid™ Premium First Strand cDNA synthesis kit (Fermentas, Thermo Scientific, Waltham, MA, USA).

qPCR was performed to analyse the expression of each gene on a Rotor gene-3000A instrument (Qiagen, Venlo, Netherlands) using the SensiFAST SYBR No-ROX Kit (Bioline, London, UK) according to the manufacturer’s instructions. Ten μL SensiFAST SYBR, 1 μL (1 μM final concentration) each forward and reverse primers, and 6 μL DNase-free water was added to 2 μL cDNA for amplification in a total volume of 20 μL. The cycling conditions were as follows: initial denaturation for 2 min at 95 °C followed by amplification and quantification cycle repeated 40 times each consisting of 5 s denaturing at 95 °C, 10 s annealing at primer specific temperatures, and 20 s extension at 72 °C. Two biological replicates were used with two technical replicates of each.

The relative standard curve method^51^ was used to quantify the expression levels of the selected genes and the data normalised using *actin* (ref.^6^ and Supplementary Fig. 12) as reference gene. Data sets were statistically compared between samples at each time point using one-way analysis of variation (ANOVA) with the statistical analysis software GraphPad InStat v3 (GraphPad software, San Diego, CA, USA). The confidence level of all analyses was set at 95%, and values with *p* < 0.05 were considered significant.

## Supplementary information

Time-resolved decoding of metabolic signatures of <Emphasis Type="Italic">in vitro</Emphasis> growth of the hemibiotrophic pathogen <Emphasis Type="Italic">Colletotrichum sublineolum</Emphasis>

## Data Availability

The study design information, LC-MS raw data, analyses and data processing information, and the meta-data have been deposited to the EMBL-EBI metabolomics repository – MetaboLights database (10.1093/nar/gks1004. PubMed PMID:23109552)^52^ with the identifier (accession number) MTBLS735 (http://www.ebi.ac.uk/metabolights/MTBLS735).
