# Supplementary material for: Time-resolved decoding of metabolic signatures of in vitro growth of the hemibiotrophic pathogen Colletotrichum sublineolum
Source: Sci Rep. 2019 Mar 1;9:3290. doi: 10.1038/s41598-019-38692-7 (PMC6397173; doi:10.1038/s41598-019-38692-7)
Supplement: Supplementary file 1 — Time-resolved decoding of metabolic signatures of in vitro growth of the hemibiotrophic pathogen Colletotrichum sublineolum [file 41598_2019_38692_MOESM1_ESM.pdf]

Scientific Reports

Supplementary File

Time-resolved decoding of metabolic signatures of *in vitro* growth of the hemibiotrophic pathogen *Colletotrichum sublineolum*

Fidele Tugizimana[1], Arnaud T. Djami-Tchatchou[1], Johannes F. Fahrmann[2], Paul A. Steenkamp[1], Lizelle A. Piater[1] and Ian A. Dubery[1,*]

[1] Department of Biochemistry, University of Johannesburg, Auckland Park, Johannesburg, South Africa
[2] Departments of Clinical Cancer Prevention, University of Texas MD Anderson Cancer Center, 1515 Holcombe Blvd., Houston, TX 77030, USA
* Author to whom correspondence should be addressed; e-mail: idubery@uj.ac.za; Tel.: +27-11-559-2401.

Supplementary Figures 1 – 12

Supplementary Table 1

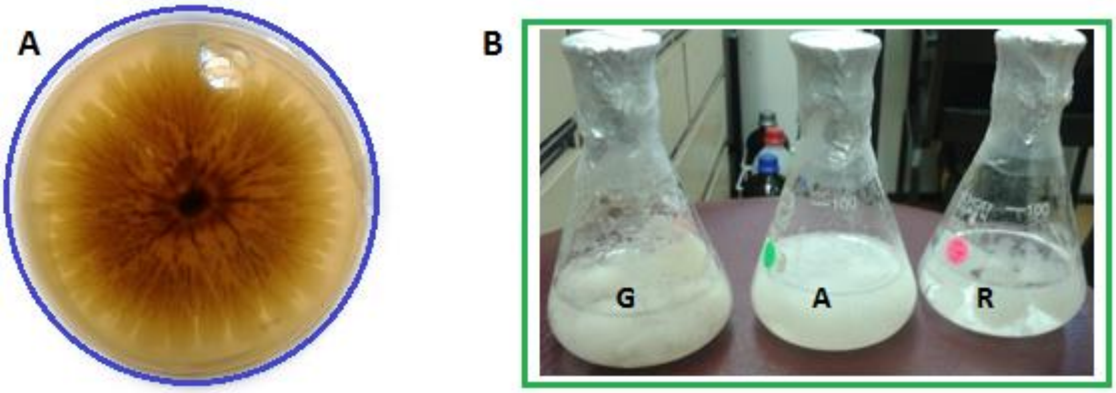

Supplementary Figure 1A, B. Cultivation of *C. sublineolum*: (A) the fungus grown on half-strength PDA plate – fungal mycelia can be seen as the fungus spreads on the plate. **(B)** *C. sublineolum* cultured in liquid MS media containing different carbon sources: **G** – glucose; **A** – arabinose; and **R** – rhamnose. The three flasks were inoculated the same day, and as it can be seen, the rate of the *C. sublineolum* growing in rhamnose-containing media (**R**) was slow compared to the other two media.

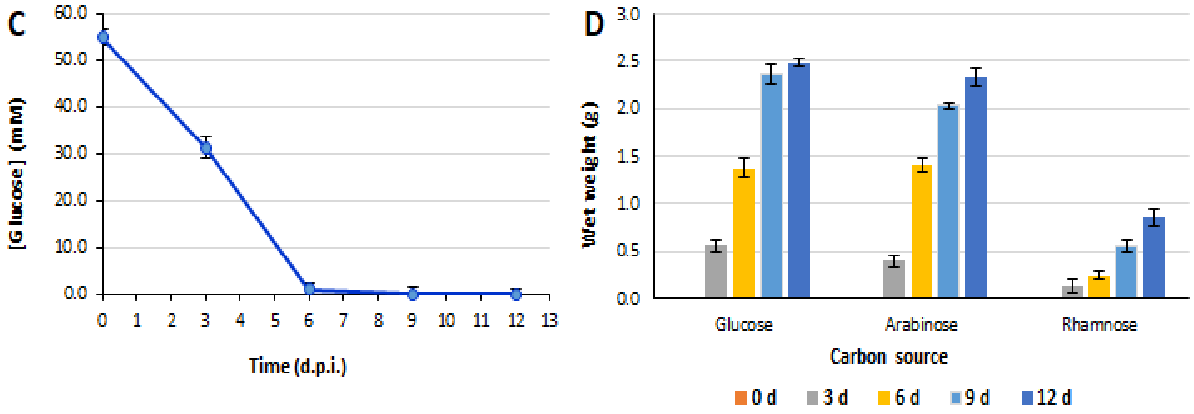

Supplementary Figure 1C, D: Assessment of the growth of the *C. sublineolum* and utilisation of carbon source. The fungus was cultured in liquid MS media containing 10 g L^{-1} of (i) glucose, (ii) arabinose and (iii) rhamnose. **(C) Glucose consumption –** glucose concentration in the media was measured at each time point as an estimate of the carbon utilisation rate. The error bars indicate standard deviation (SD). Each sample was measured twice and the data represent the average of three independent experiments. **(D) *C. sublineolum* biomass accumulation –** the wet-weight-based estimation of the growth of the fungus over 12 days. The filtered mycelia were weighed and the mass recorded at each time point. The experiment was repeated three times. The error bars indicate standard deviation (SD).

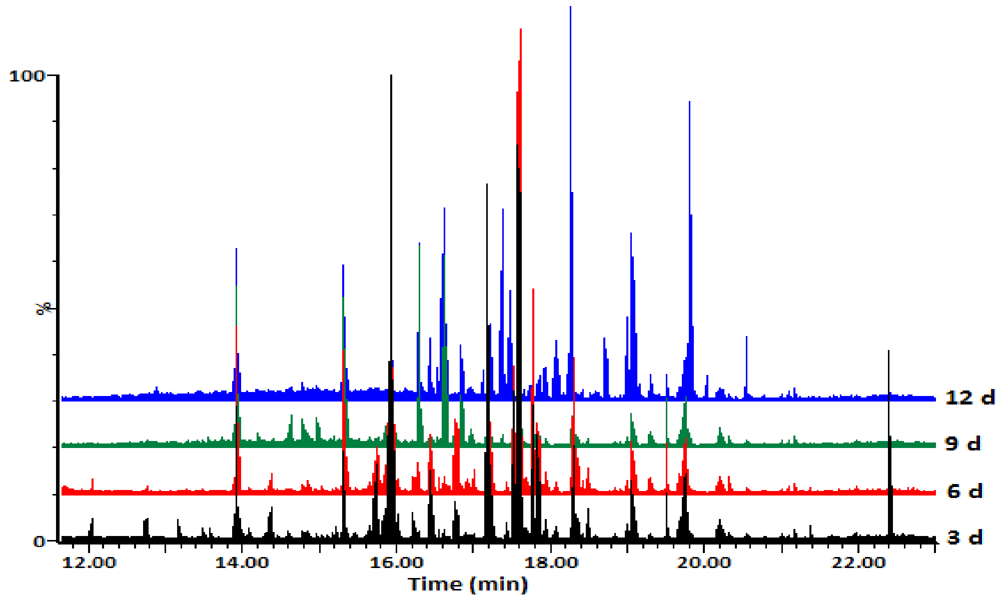

Supplementary Figure 2: UHPLC-MS BPI chromatograms of peak intensity (ESI negative mode) *vs.* Rt of <u>intracellular</u> extracts of *C. sublineolum* grown on <u>glucose</u> as carbon source. Extracts correspond to samples of *C. sublineolum* sub-cultured in glucose-containing liquid media and harvested over time at 3, 6, 9 and 12 d.p.i.. Visually, time-related differences are observed on the chromatograms, indicating changes in the metabolome of *C. sublineolum* during growth and nutrient availability. Multivariate statistical analysis tools were used to extract and explain these observable differences.

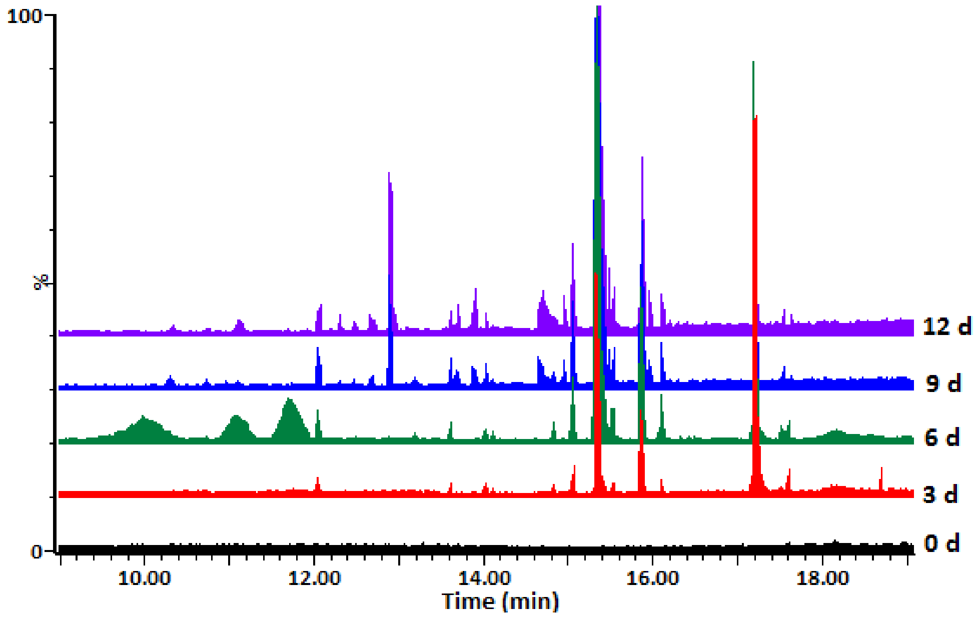

Supplementary Figure 3: UHPLC-MS BPI chromatograms of peak intensity (ESI negative mode) *vs.* Rt of <u>extracellular</u> samples of *C. sublineolum* grown on <u>glucose</u> as carbon source. Extracts correspond to samples of *C. sublineolum* sub-cultured in glucose-containing liquid media and harvested over time: 3, 6, 9 and 12 d post-inoculation. Day 0 samples refer to the glucose media prior to incubation time. Visually, time-related differences are observed on the chromatograms, indicative of metabolites secreted into the medium by *C. sublineolum* over time. Multivariate statistical analysis tools were used to extract and explain the differences observed on the chromatograms.

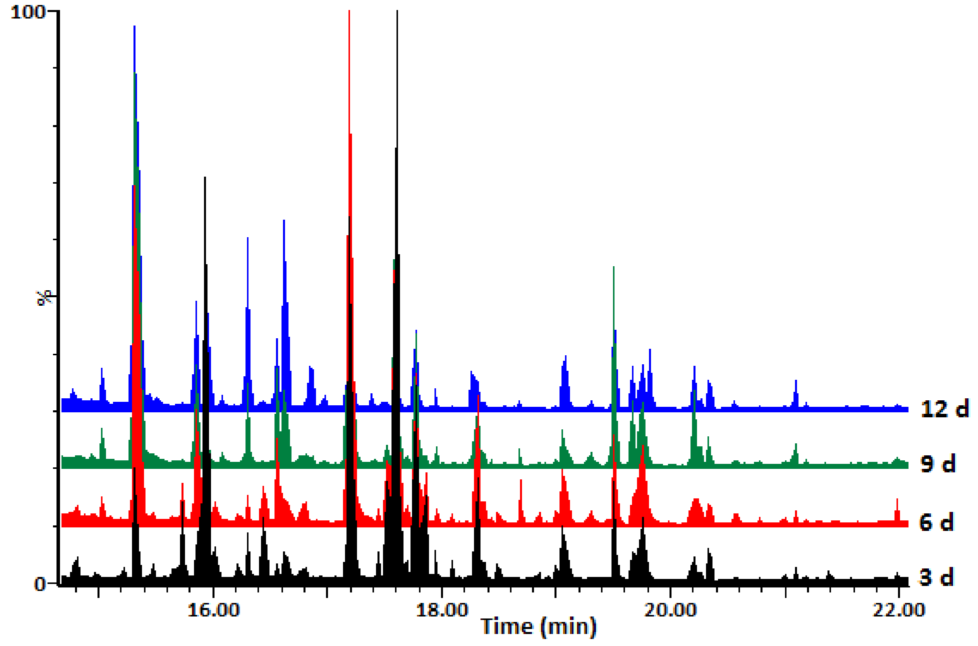

Supplementary Figure 4: UHPLC-MS BPI chromatograms of peak intensity (ESI negative mode) *vs.* Rt of <u>intracellular</u> extracts of *C. sublineolum* grown on <u>arabinose</u> as carbon source. These are samples of *C. sublineolum* sub-cultured in arabinose-containing liquid media and harvested over time: 3, 6, 9 and 12 d post-inoculation. Visually, time-related differences are observed on the chromatograms, suggesting changes in the metabolome of the *C. sublineolum* over time. Multivariate statistical analysis tools were used to extract and explain the differences observed on the chromatograms.

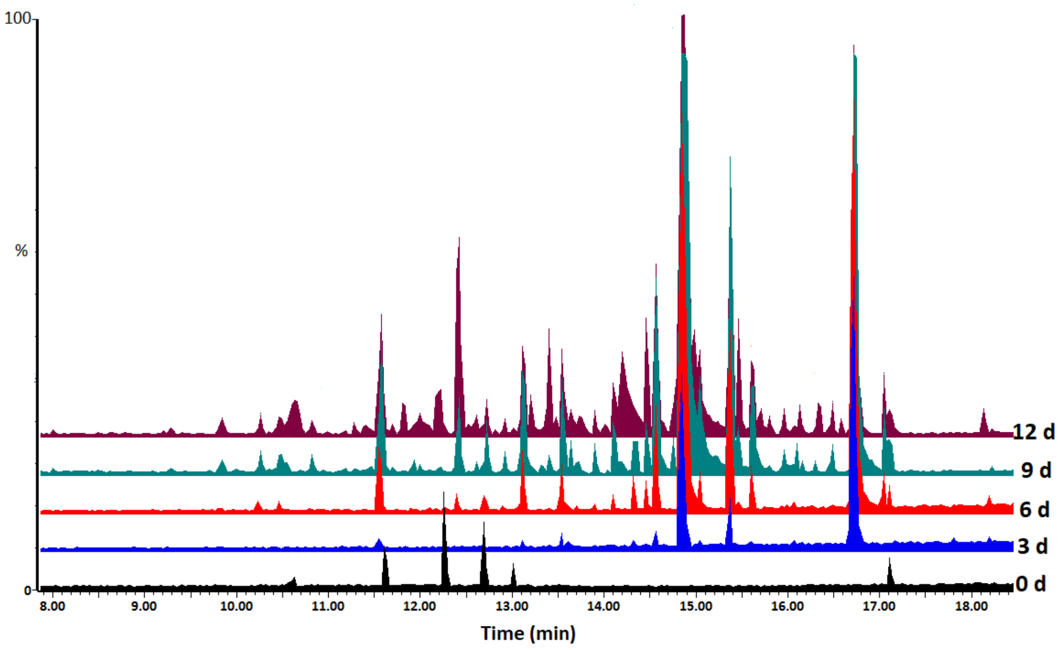

Supplementary Figure 5: UHPLC-MS BPI chromatograms of peak intensity (ESI negative mode) *vs.* Rt of <u>extracellular</u> extracts of *C. sublineolum* grown on <u>arabinose</u> as carbon source. These are samples of *C. sublineolum* sub-cultured in arabinose-containing liquid media and harvested over time: 3, 6, 9 and 12 d post-inoculation. Day 0 refers to arabinose-containing media prior to incubation time. Visually, time-related differences are observed on the chromatograms, suggesting changes in the exo-metabolome of the *C. sublineolum* over time. Multivariate statistical analysis tools were used to extract and explain the differences observed on the chromatograms.

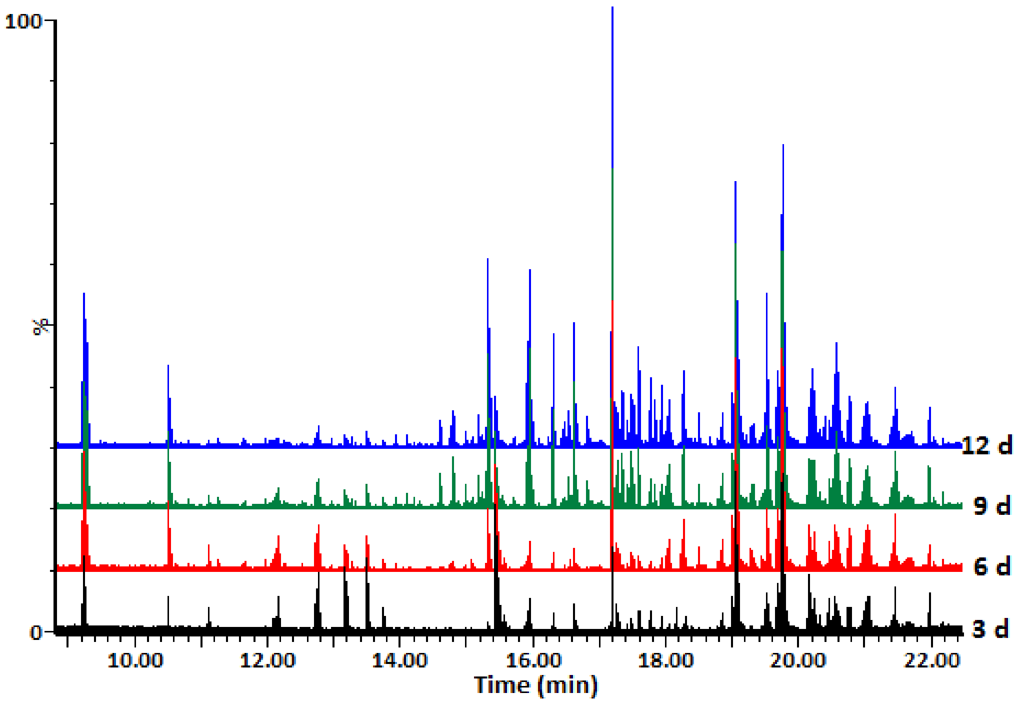

Supplementary Figure 6: UHPLC-MS BPI chromatograms of peak intensity (ESI positive mode) *vs.* Rt of <u>intracellular</u> samples of *C. sublineolum* grown on <u>rhamnose</u> as carbon source. These are samples of *C. sublineolum* sub-cultured in rhamnose-containing liquid media and harvested over time: 3, 6, 9 and 12 d post-inoculation. Visually, time-related differences are observed on the chromatograms, suggesting changes in the metabolome of the *C. sublineolum* over time. Multivariate statistical analyses tools were used to extract and explain the differences observed on the chromatograms.

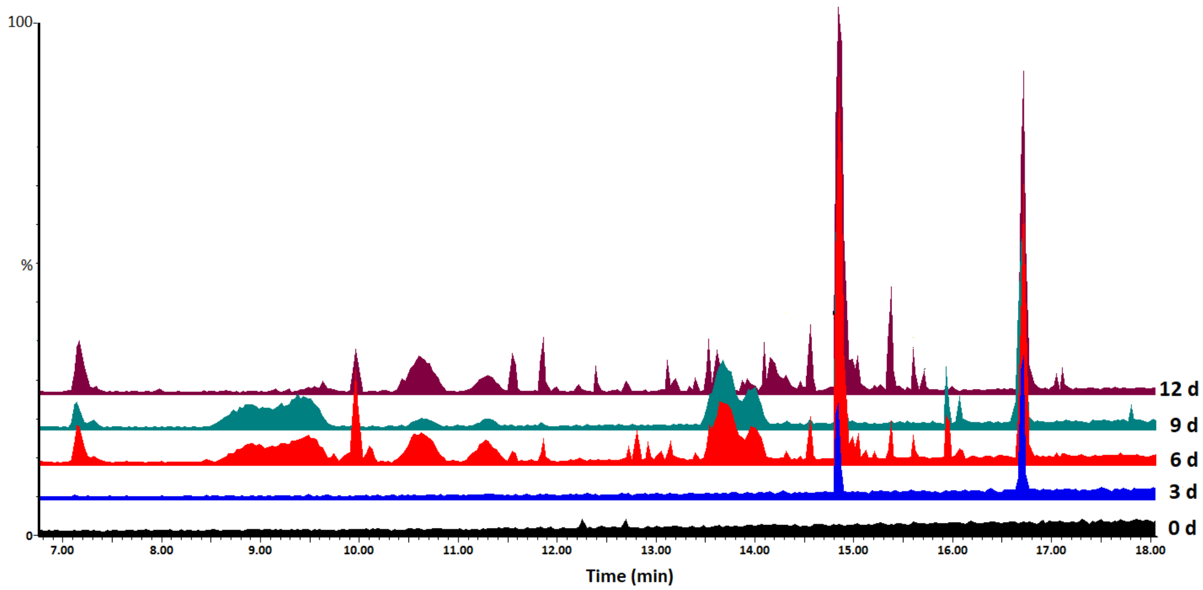

Supplementary Figure 7: UHPLC-MS BPI chromatograms of peak intensity (ESI negative mode) *vs.* Rt of <u>extracellular</u> extracts of *C. sublineolum* grown on <u>rhamnose</u> as carbon source. These are samples of *C. sublineolum* sub-cultured in rhamnose-containing liquid media and harvested over time: 3, 6, 9 and 12 d post-inoculation. Day 0 refers to rhamnose-containing media prior to incubation time. Visually, time-related differences are observed on the chromatograms, suggesting changes in the exo-metabolome of the *C. sublineolum* over time. Multivariate statistical analysis tools were used to extract and explain the differences observed on the chromatograms.

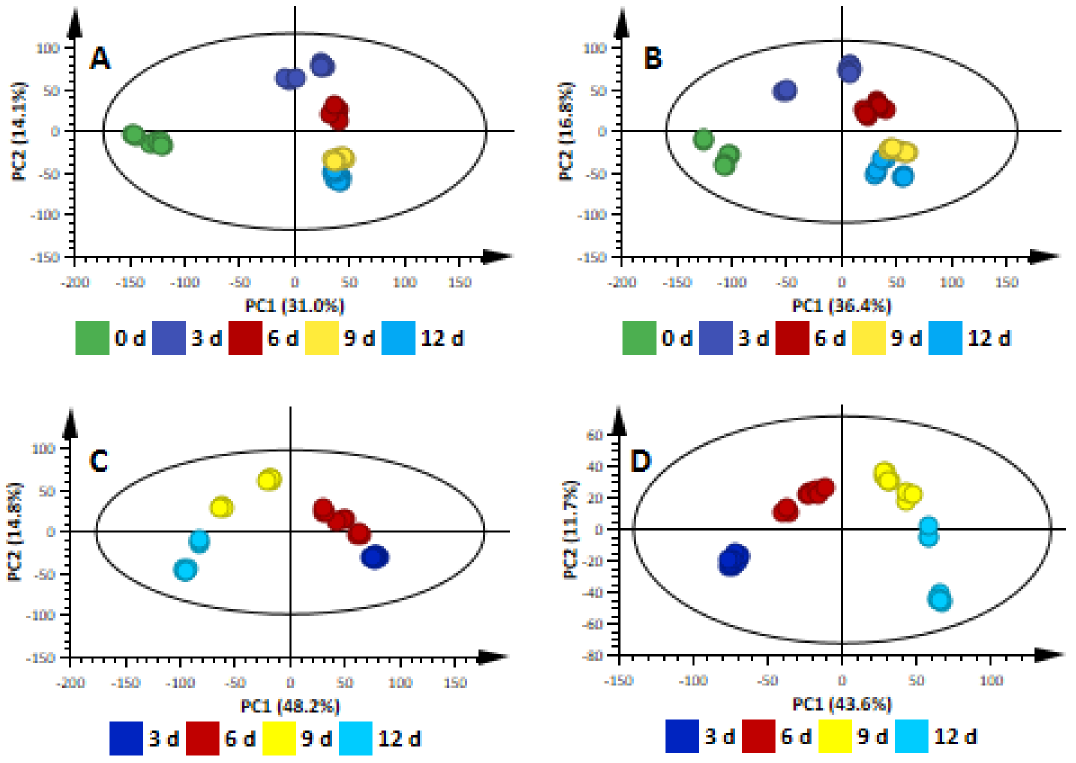

Supplementary Figure 8: **PCA modelling of data from samples of *C. sublineolum* cultured in glucose-containing MS liquid media**. (**A**) **ESI negative data** from **extracellular extracts**: a scores scatter plot of a 3-component model, explaining 53.8% of the total variation in the Pareto-scaled data, with the amount of predicted variation by the model, according to cross-validation, as 37.7%. (**B**) **ESI positive data** from **extracellular samples**: a 2D scores plots (first two PCs) of a 7-component model that explains 77.9% of the total variation in Pareto-scaled data X, with the amount predicted variation as 65.1%. (**C**) **ESI negative data** from **intracellular extracts**: PCA scores plot (first two PCs) of a 4-PCs model, with R^2 = 0.71, and Q^2 = 0.617 (according to cross-validation). (**D**) **ESI positive data** from **intracellular samples**: PCA scores plot (first two PCs), of a 3-component model, explaining 60.3% of the total variation in Pareto-scaled data X, with the amount of predicted variation by the model, according to cross-validation, as 51.5%. All the computed PCA models (**A**-**D**) show time-related sample clustering in the scores space, with clear time trends and less variation within-group.

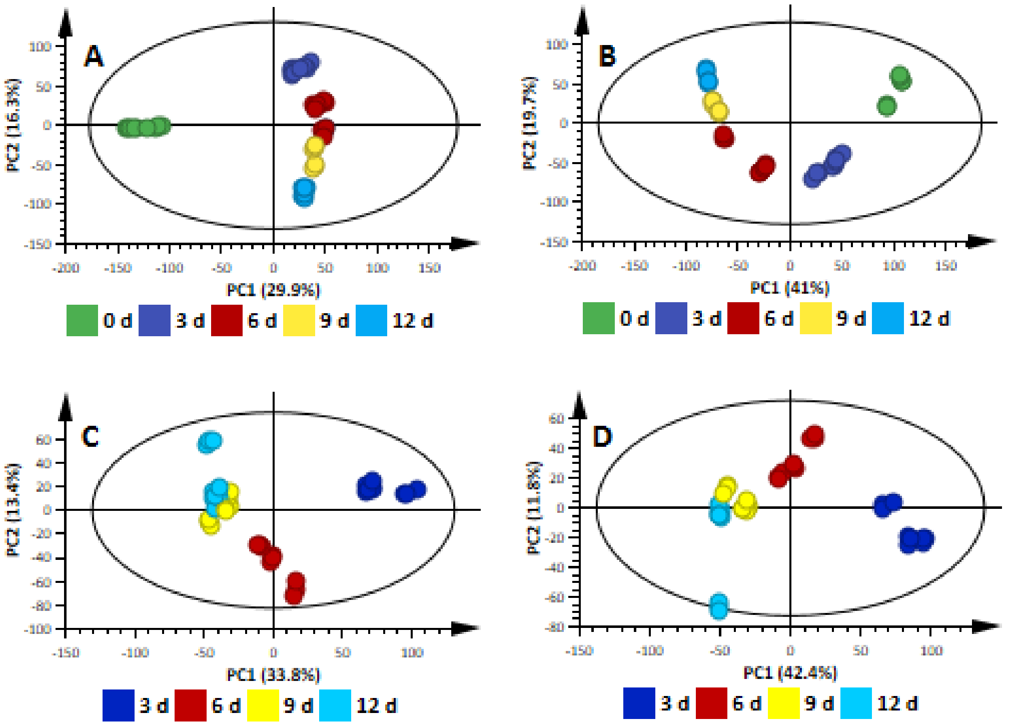

Supplementary Figure 9: **PCA modelling of data from samples of *C. sublineolum* cultured in rhamnose-containing MS liquid media**. (**A**) **ESI negative data** from **extracellular extracts**: a scores scatter plot of a 3-component model, explaining 53.3% of the total variation in the Pareto-scaled data, with the amount of predicted variation by the model, according to cross-validation, as 43.8%. (**B**) **ESI positive data** from **extracellular samples**: a 2D scores plots (first two PCs) of a 7-component model that explains 80.9% of the total variation in Pareto-scaled data X, with the amount predicted variation as 71.2%. (**C**) **ESI negative data** from **intracellular extracts**: PCA scores plot (first two PCs) of a 3-PCs model, with $R^2 = 0.579$, and $Q^2 = 0.45$ (according to cross-validation). (**D**) **ESI positive data** from **intracellular samples**: PCA scores plot (first two PCs), of a 4-component model, explaining 66.2% of the total variation in Pareto-scaled data X, with the amount of predicted variation by the model, according to cross-validation, as 53.2%. All the computed PCA models (**A**-**D**) show time-related sample clustering in the scores space, with clear time trends and less variation within-group.

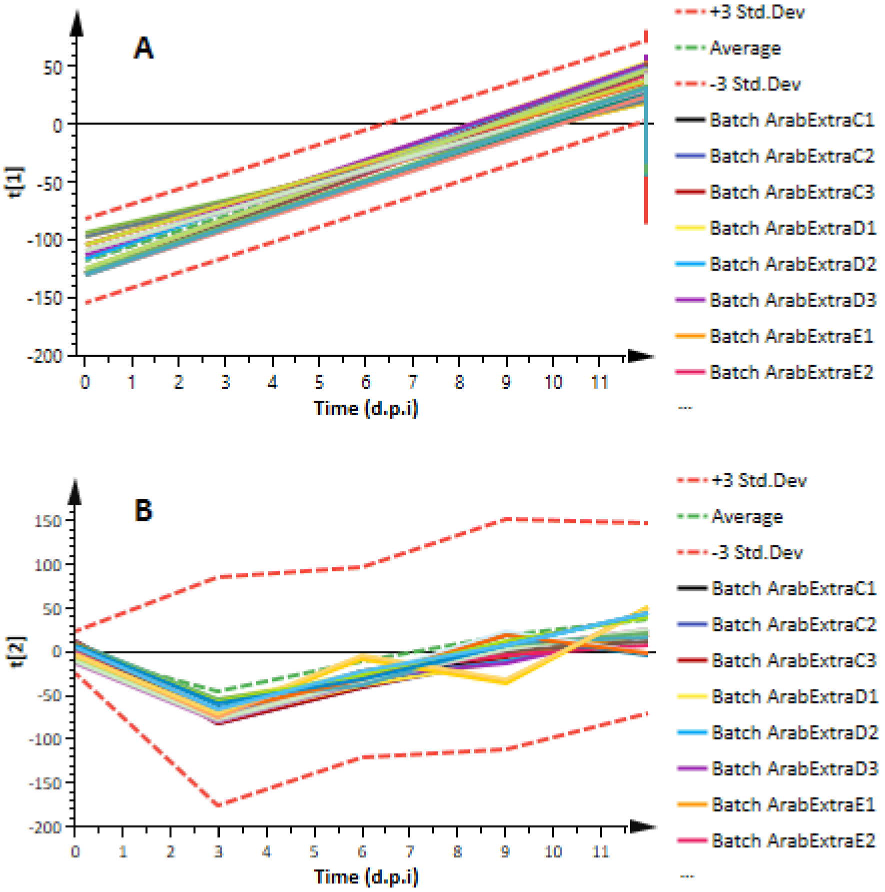

Supplementary Figure 10: PLS trajectory plots from batch evolution modelling. (A) ESI positive data of extracellular samples from *C. sublineolum* grown on different carbon sources (arabinose, glucose and rhamnose) for 3, 6, 9 and 12 d.p.i. The computed PLS model of six significant components explaining 67.2% of the variation in the Pareto-scaled data X and 98.5% of the variation in the response Y (time). The predicted variation by the model, according to cross-validation, is 97.3%, indicating a significant relationship with time. CV-ANOVA *p*-value = 0. The model describes the metabolic changes over time. (**B**) Lower scores vector t[2] of the PLS-model in **Figure 2A**. The vector t[2], orthogonal to the first vector t[1], explains less of the covariance (compared to the first vector) in the model. Such a plot can aid in assessing more subtle time-related metabolic variations; which can be observed in some samples (with directional changes over time). Despite some small nuances, the trajectory changes extracted and described by these PLS-models all pointed to clear time trend patterns, indicating major metabolic perturbations to be in the periods of 0-3, 3-6 and 6-12 d.p.i.

Background: Extracts from intracellular and extracellular (secreted) fungal samples were analysed via UHPLC-MS. Following principal component analyses (PCA), partial least squares (PLS) based batch processing/modeling (BP/M) analysis was used to interpret (mine) the data, treating each carbon source (glucose, arabinose and rhamnose) as an individual batch, comprising a series of times (3, 6, 9,12 d.p.i. samples). BP/M provided an efficient means of infographically visualising the biochemical response to the type of carbon source in terms of both inter-group variation and net variation in intra/extracellular metabolite profiles. BP/M allowed for the evolution of the metabolic changes to be statistically characterised and described in time trajectories and provided thus a template for defining the sequence of time-dependent metabolic profiles.

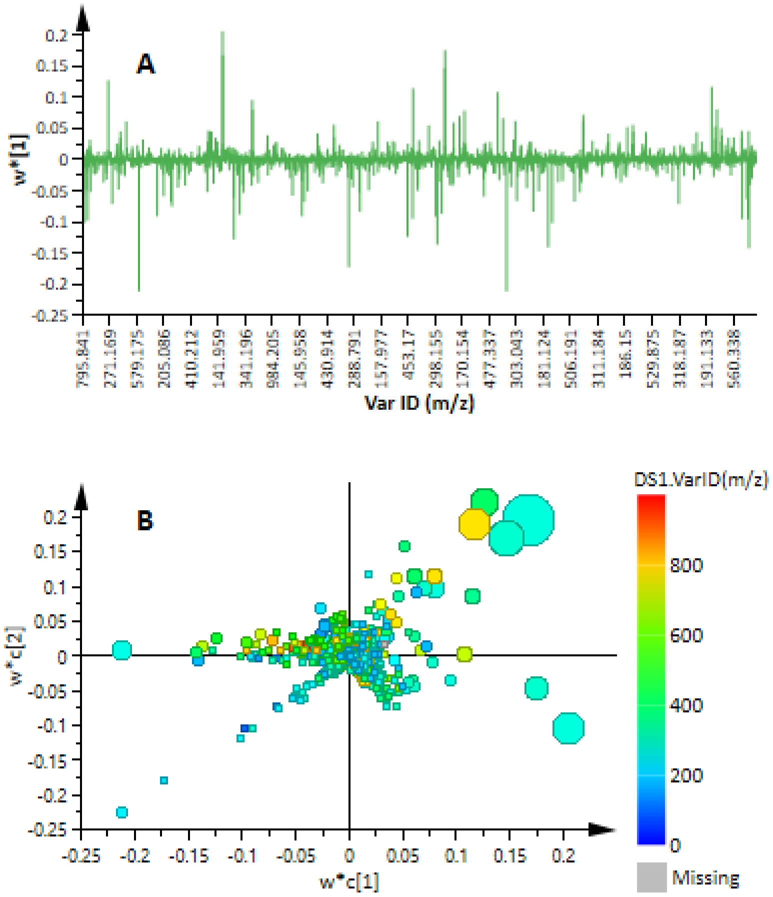

Supplementary Figure 11: Representative infographics of variable selection in the batch modelling, PLS-loadings plots. (A) A line plot of the loading vector for the first component of the PLS-model in **Fig. 2A**. The plot allows the identification of the variables (positively and negatively correlated features) that contribute to the significant time-related changes observed in the scores trajectory plot. **(B)** A scatter loading plot of the PLS-model in **Fig. 2B**. The loadings are colour-coded based on the *m/z* range (100–1000 Da), and sized based on the time point, 3 d.p.i.: the bigger the size of the loading (bubble), the more important it is for time-related alterations at 3 d.p.i. These typical loadings plots allowed the identification of the variables that explained the time-dependent variation, described by the trajectory plots.

.

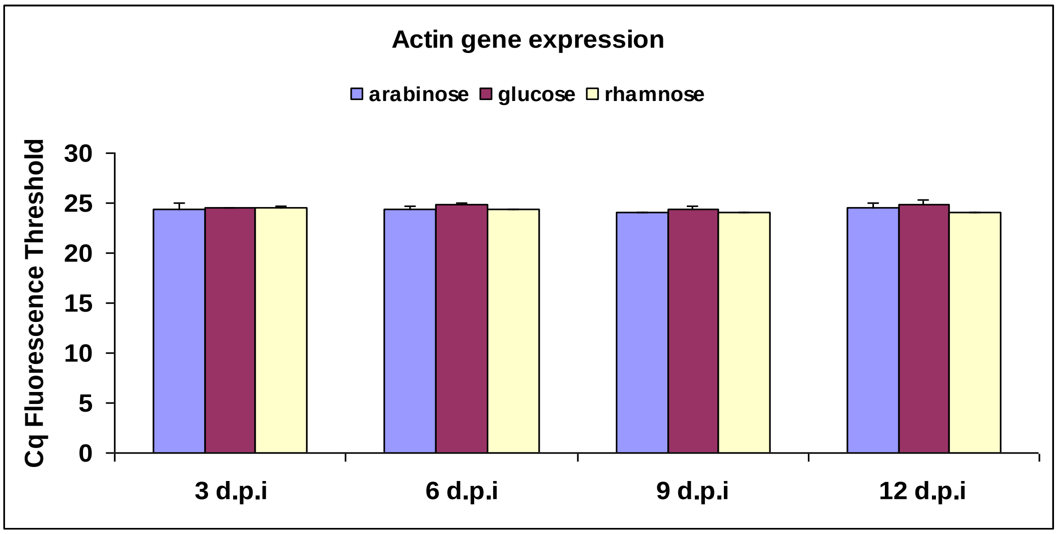

Supplementary Figure 12: Gene expression analysis of actin gene in *C. sublineolum* grown in MS media containing either glucose (A), rhamnose (B) or arabinose. Equivalent amounts of RNA were used in each reaction, and data points represent the raw data for the *Colletotrichum sublineolum actin* gene. Data are not normalized to a reference gene and instead are graphed as the (Cq) value, which is proportional to the Log2 expression level of the *actin* gene. Error bars represent standard error of the mean. In addition the cycle threshold values were analysed for expression stability at each time point using NormFinder PCR analysis software and the stability values ranged between 0.001and 0.019 (data not shown).

Supplementary Table 1: Base composition of primers designed for the selected genes investigated using qPCR.

Genes	Primer Sequence (5'-3')		Amplicon length (bp)
Zinc carboxypeptidase (JMSE01000325.1)	CCCTCACGTACCAAATCATC	Forward	93
	CGATGGGCAGAATGTAGAAG	Reverse	
Putative sodium/hydrogen exchanger family protein (JMSE01001189.1)	CCTTGAGACTTGGCTCTATTT	Forward	99
	TTTCTCCTGACAGCGAATTT	Reverse	
GMC oxidoreductase (JMSE01001299.1)	GAGTGTCTCGCTCCAAATAC	Forward	109
	GACTGACCACGTCCATAATC	Reverse	
Fungal cellulose binding domain-containing protein (JMSE01001209.1)	CAGTTCATCCGCGACTTTA	Forward	98
	CCATCAACGACAGAGTTGT	Reverse	
Pectate lyase (JMSE01001554.1)	CCTCAAGAACGCCATCATC	Forward	81
	CACCAGACGTTCTGAATGG	Reverse	
Putative peptidase family M28 (JMSE01001300.1)	ATCCACACCTCCAGAGATAC	Forward	101
	CCCAAGTAAGAAGCCTCAAC	Reverse	
Actin (JQ005834.1)	AGATTCGGTCACAGATTGTC	Forward	146
	CTATTCACATGTTAGGTTGAAGC	Reverse	

Based on the putative function during biotrophic and necrotrophic development of *Colletotrichum* species (O'Connell et al., 2012, reference 6), six genes were selected for gene expression analysis in *C. sublineolum* grown in a culture containing either arabinose, glucose or rhamnose as carbon source. The primer pairs were designed using the 'Primer Quest' tool (Integrated DNA Technologies, Coralville, IA, USA) from *C. sublineolum* sequences obtained in Genbank (www.ncbi.nlm.nih.gov/genbank).
